# Wavelength Characteristics and Visual Function of Photochromic Contact Lenses in Indoor and Outdoor Conditions

**DOI:** 10.3390/jcm12237417

**Published:** 2023-11-30

**Authors:** Shuya Suzuki, Kazutaka Kamiya, Tatsuya Iizuka, Tomoya Handa

**Affiliations:** 1 Graduate School of Medical Science, Kitasato University, Sagamihara 2520373, Kanagawa, Japan; suzuki.shuya06@gmail.com (S.S.);; 2 Visual Physiology, School of Allied Health Sciences, Kitasato University, Sagamihara 2520373, Kanagawa, Japan; 3 Department of Rehabilitation, Orthoptics and Visual Science Course, School of Allied Health Sciences, Kitasato University, Sagamihara 2520373, Kanagawa, Japan

**Keywords:** photochromic CL, wavelength, indoor, outdoor, glare

## Abstract

Purpose: To examine the wavelength characteristics of photochromic contact lenses (CL) and evaluate the impact of tinting on visual function in indoor, outdoor, and glare environments. Methods: A total of 33 healthy individuals with refractive errors were recruited and fully corrected for refractive errors. Three groups were established, including non-activated photochromic CL, activated photochromic CL, and lenses without photochromic properties, which replicated the dimming characteristics of CL. Visual acuity and contrast sensitivity were measured and compared among the three groups. Results: Statistically significant differences were observed in the spatial frequency (6, 12 cpd) and contrast sensitivity outdoors, with improved values recorded in the activated photochromic-CL group. In subsequent comparisons, the activated-photochromic-CL group demonstrated significantly better contrast-sensitivity values than the non-photochromic-CL group, as well as significant improvement in contrast sensitivity compared to the non-activated-photochromic-CL group. No significant differences were observed in the indoor or outdoor visual acuity. Conclusion: Our results suggest that photochromic CL enhances visual function in outdoor environments, while maintaining visual function indoors and under glare, thereby improving the quality of vision (QOV) in severe light conditions where exposure to sunlight and ultraviolet light is anticipated.

## 1. Introduction

Our eyes are subjected to high-energy radiation in the form of ultraviolet and blue light from various sources, including the Sun, on a daily basis. Such exposure has been shown to be a potential strain on eye health and may result in eye conditions such as cataracts and pterygium [1,2]. Ultraviolet radiations (UVRs) are the most energetic part and account for most of the damage caused by sunlight. The crystalline lens and cornea absorb most of the UVR that enters the eye and are, therefore, particularly vulnerable to damage from sunlight [3,4]. Additionally, everyday vision can also be impaired by visual aberrations such as glare, halos, starbursts, and light scattering, leading to impairment of visual performance and the onset of headaches [5]. According to the International Commission on Illumination’s (CIE’s) definition, unpleasant glare causes visual irritation or discomfort without necessarily impairing vision [6]. Light-shielding and polarizing lenses have been used in an attempt to mitigate glare; however, these lenses absorb visible light peaks, resulting in the impairment of visual function [7]. The light-shielding and polarizing lenses have disadvantages over photochromic lenses in term of the difficulty of driving at night due to low spectacle transmission and the need for putting on and taking off in daily life. Corning developed photochromic glasses that modulate the cutoff rate of wavelengths based on the intensity and wavelength of light [8]. A dimmer is a molecule that is reversibly isomerized into a molecule with a different absorption spectrum depending on a specific wavelength. In the absence of light of a specific wavelength to activate it, it returns to its original molecular structure and fades accordingly. This inactivation process is also affected by the temperature. Therefore, even if light of a specific wavelength is eliminated, when the temperature of the environment is low, the process of non-activation does not proceed, and the color will not fade. Since the CL is located on the surface of the eye and its temperature is constant, it is possible to provide a specific environment where the sensitivity of the dimmer is less affected by the temperature of the environment. ACUVUE^®^ OASYS with Transitions (Johnson & Johnson Vision, Jacksonville, FL, USA) is a new type of photochromic CL that modulates its wavelength characteristics based on UVR, generating interest in its functional characteristics. Photochromic CLs do not cut off all wavelengths, as is the case with ordinary sunglasses, but rather vary the cutoff rate according to the wavelength. It has been recently reported that partially cutting certain wavelengths (420 nm or less, 460 nm, and 585 nm) improves contrast-sensitivity function [9]. It is indicated that the photochromic CLs naturally enhance visual quality due to the smooth transition in the tone of color. However, the effects of the wavelength characteristics of these lenses have not been fully investigated because of the limitations of visual-function testing performed only under indoor conditions. Visual function should also be assessed under bright sunlight and glare conditions, since photochromic CLs were expected to be mostly beneficial. However, there have been no studies on the visual function of photochromic CLs under such conditions. Considering that the degree of discoloration varies with body temperature, air temperature, and UVR dose, it is difficult to unify the fully activated and deactivated states of the photochromic CL, making it impossible to accurately measure the effect of the wavelength characteristics of the photochromic CL on visual function. For example, photochromic CLs return to the deactivated state faster at higher temperatures, resulting in variations in these wavelength characteristics [10]. As far as we can ascertain, this is the first pilot study to simulate the visual function of photochromic CLs under bright sunlight and glare conditions. Indeed, we have actually used ophthalmic lenses in this study, since it is highly difficult to unify the wavelength characteristics of photochromic CLs, based on the fact that the UVR exposure and the temperature have influenced these characteristics.

## 2. Materials and Methods

**Figure 1:**
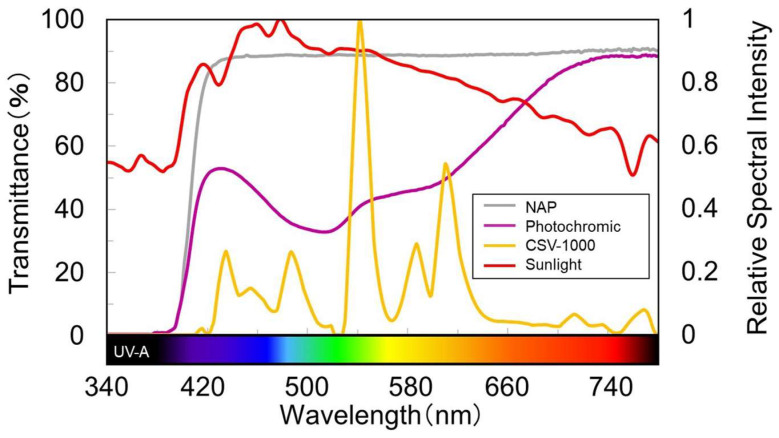
Spectral transmittance curve of simulated lens reproducing wavelength characteristics of photochromic CL. NAP: Non-activated photochromic CL.

**Figure 2:**
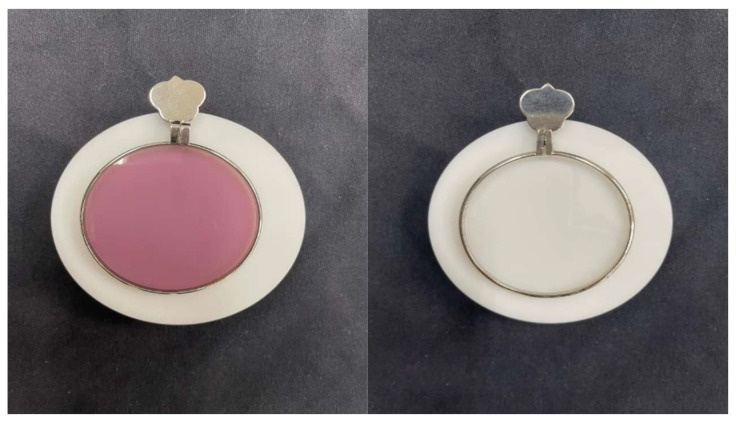
Simulated lens taken with a D65 light source on a white plate (**left**: photochromic lens in activated state, **right**: photochromic lens in deactivated state).

University students aged 18 years or older who had no ocular diseases other than refractive errors (spherical errors of −8 to 2 D and cylindrical errors of 0 to 1.5 D) were eligible for inclusion in the current study. A total of 33 eyes of 33 participants (mean age 21.3 ± 1.2 years; 13 male and 20 female) were enrolled for this study. The sample size in this study offered 87.8% statistical power at the 5% level when the effect size was 0.5. We randomly measured only one eye for each subject for data analysis. The study was conducted from July 2022 to October 2022 in accordance with the Declaration of Helsinki. Prior to initiation, the objective of the study was fully communicated to the participants and written informed consent was obtained from all participants. The study was conducted in accordance with the principles of the Declaration of Helsinki and approved by the Ethics Committee of the School of Allied Health Sciences at Kitasato University (2022-006). Written informed consent was obtained from all participants in accordance with our Institutional Review Board protocol, which involved randomizing the left and right eyes of the participants and conducting a comprehensive refractive-correction examination using an optometric frame in three different environments: indoors (illumination of 600 lx), outdoors (under direct sunlight with a horizontal illumination of 89,508 ± 6466 lx and a vertical illumination of 84,343 ± 6596 lx), and under glare conditions. The outdoor measurements were conducted under bright sunlight conditions between 11 to 14 o’clock on a sunny summer day between August and September. We confirmed that outdoor illuminance was 80,000 lx or more in all cases. Illuminance was measured using an illuminance meter (CL-200A, KONICA MINOLTA, Inc., Tokyo, Japan). The examination under direct sunlight was adjusted such that the sun was positioned in front of the participant. Participants wore ophthalmic lenses in the deactivated state and in the activated state after full refractive correction in order to simulate the wavelength characteristics of the ACUVUE^®^ OASYS with Transitions^TM^ (Figure 1 and Figure 2) on the front of the optometric frame. Lenses with reproduced wavelength characteristics were checked and verified for transmittance of real photochromic CLs using CIE standard illuminant D65 (SOLAX-iO; SERIC Ltd., Saitama, Japan) and an illuminance meter according to ISO [11]. This study comprised three groups: the non-photochromic-lens group, non-activated-photochromic-CL group, and activated-photochromic-CL group. The following items were evaluated and compared.

### 2.1. Visual Acuity

Visual acuity was assessed at 5 m using a visual acuity meter (NON-GLARE VISION CHART VC-22^®^; TAKAGI, Nagano, Japan). Outdoor visual acuity was evaluated using a single-character optotype (Landolt ring-alone optotype, Handaya, Tokyo, Japan).

### 2.2. Contrast Sensitivity

Contrast sensitivity was evaluated indoors (illuminance of 600 lx), outdoors (direct sunlight, horizontal illuminance: 89,508 ± 6466 lx, vertical illuminance: 84,343 ± 6596 lx), and under glare conditions at a viewing distance of 2.5 m using a contrast-sensitivity measuring instrument (CSV-1000^®^, Vector Vision, Greenville, SC, USA) [12]. The glare was provided by the built-in glare light of the CSV-1000. Participants were asked to indicate the direction of the lightest stripe in each frequency range, and the area under the log contrast-sensitivity function (AULCSF) was quantitatively compared using the AULCSF calculated according to Applegate et al. [13]. In brief, the log of contrast sensitivity was plotted as a function of log spatial frequency, and third-order polynomials were fitted to the data. The fitted function was integrated, and the resultant value was expressed as the AULCSF.

Three orders of testing (both the measurement environment and the ophthalmic lens) and the selection of the test eye (right or left eye) were performed in a randomized fashion. The measurement interval was set at 3 min in each subject.

Statistical analysis was performed using the analysis software BellCurve for Excel version 4.03 (Social Survey Research Information Co., Ltd., Tokyo, Japan). If significant, multiple comparisons were performed using the Tukey–Kramer test.

## 3. Results

**Figure 3:**
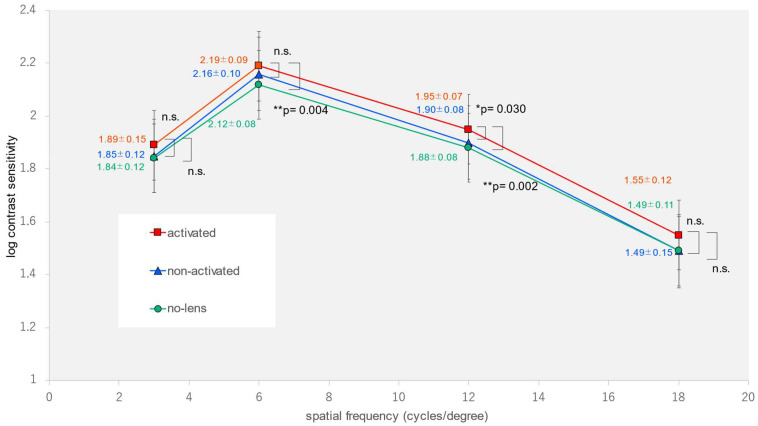
Contrast sensitivity at each spatial frequency outdoors using * *p* < 0.05 ** *p* < 0.01 and Tukey–Kramer test.

**Figure 4:**
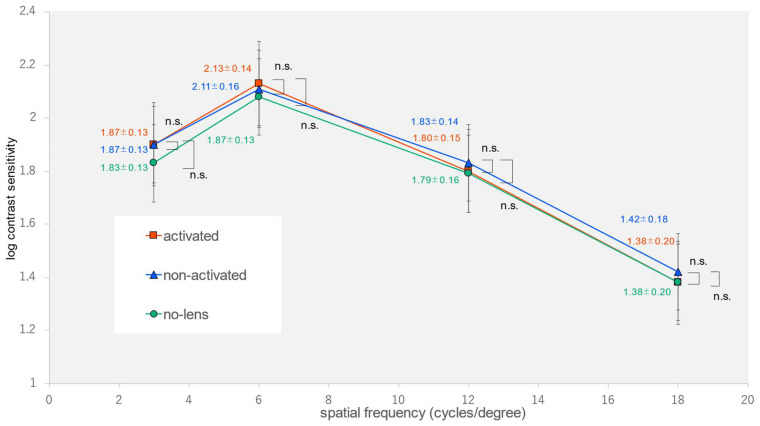
Contrast sensitivity at each spatial frequency indoors.

**Figure 5:**
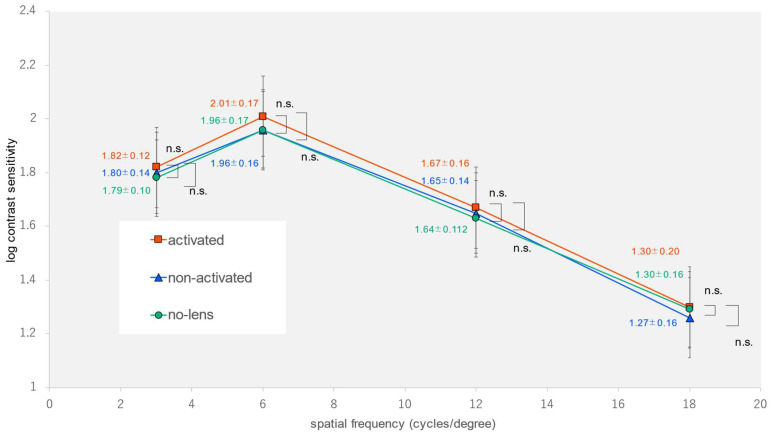
Contrast sensitivity at each spatial frequency under glare.

The demographic characteristics of the participants are summarized in Table 1. The spherical error was measured as −1.80 ± 2.82 diopters, while the cylindrical error was found to be 0.64 ± 0.59 diopters. We found statistically significant differences in the spatial frequencies (6 and 12 cycles per degree) and the AULCSF outdoors in the activated-photochromic-CL group compared to the non-photochromic-lens group and the non-activated-photochromic-CL group (one-way analysis of variance, *p* = 0.005, *p* = 0.002, *p* < 0.001) (Table 2) (Figure 3). Multiple comparisons showed a significant improvement in the two spatial frequencies (6 and 12 cycles per degree) and the AULCSF in the activated-photochromic-CL group compared with the non-photochromic-lens group, as well as a significant improvement in the spatial frequency (12 cycles per degree) and the AULCSF when compared with the non-activated CL group (Tukey-Kramer test, *p* = 0.004, *p* = 0.002, *p* < 0.001, *p* = 0.030, *p* = 0.014). In contrast, we found nonsignificant differences in terms of visual acuity under indoor and outdoor conditions; remaining spatial frequencies (3 or 18 cycles per degree) under indoor, outdoor, or glare conditions; and AULCSF under indoor or glare conditions (one-way analysis of variance, *p* = 0.949, *p* = 0.540, *p* = 0.080, *p* = 0.313, *p* = 0.414, *p* = 0.599, *p* = 0.101, *p* = 0.696, *p* = 0.355, *p* = 0.281) (Figure 4 and Figure 5).

## 4. Discussion

A summary of prior evaluations of the photochromic CL is presented in Table 3. However, studies [14] that investigate the performance of photochromic CLs in both indoor and outdoor environments are lacking, except for a single examination. Furthermore, the number of studies that have gauged other visual functions, in addition to subjective assessment by test subjects, remains limited. As far as we can ascertain, this is the first pilot study to simulate visual function of photochromic CLs under bright sunlight and glare conditions. Indeed, we have actually used optometric lenses in this study, since it is highly difficult to unify the wavelength characteristics of photochromic CLs, based on the fact that the UVR exposure and the temperature have influenced these characteristics. Hence, to better understand the impact of photochromic CL on visual performance in indoor environments, outdoor environments (under blight sunlight), and glare conditions, visual acuity and contrast sensitivity were assessed using spectacle lenses simulating the wavelength characteristics of photochromic CLs. This examination focused on the effects of the wavelength characteristics of the photochromic CL on visual function.

The present study found the effects of the wavelength characteristics of photochromic CLs on visual function outdoors under the bright sunlight, indoors, and under glare, with the wavelength characteristics perfectly unified. The current findings revealed a statistically insignificant difference in contrast sensitivity within indoor environments, but only for the activated-photochromic-CL group outdoors under the bright sunlight, suggesting that photochromic CLs might enhance visual performance in outdoor settings while preserving visual acuity within indoor environments. Therefore, these lenses may be ideal for individuals who experience photophobia but do not wish to wear tinted lenses or glasses.

The demand for photochromic CLs is higher outdoors, particularly in outdoor activities such as driving and sports, as sunlight scatter within the human eye reduces retinal image contrast [15]. Our finding indicates that photochromic CLs in the activate state may improve visual quality even under bright sunlight conditions. Based on our simulation study, we believe that photochromic CL is one of the viable options for enhancing visual quality under such tough conditions. The color tone of photochromic CL changes under ultraviolet light, but it is believed that the color-tone change within vehicles would be minimal because of the car windshield’s ability to block ultraviolet light. However, Hirata [16] reported substantial color tone alteration in all 12 vehicle types, as the functionality of the photochromic CL was fully demonstrated during daytime driving. However, it takes over 20 min for the photochromic CL to return to its inactive state once activated, and the color tone changes during this period [10]. This has raised concerns that driving might be impaired when entering a tunnel, given that lenses are worn after color tone alteration in dark surroundings. Nevertheless, the current results indicated a statistically insignificant difference in contrast sensitivity under glare conditions, with no decrease in visual acuity and contrast sensitivity for lenses in the activated-photochromic-CL group, suggesting that these lenses may be used for tunnel driving without any visual performance degradation. Moreover, halos and starbursts have been reported to decrease even when the photochromic CL is not activated, such as during nighttime [17], indicating that wearing lenses throughout the day may not pose a problem.

Several reports have also indicated that photochromic CLs can improve visual function in non-activated conditions in indoor environments. Kamiya et al. reported an improvement in dynamic and functional visual acuity and subjective satisfaction when wearing photochromic CLs indoors [18], while Hammond et al. reported improvements in discomfort under glare and time to visual function recovery after 5 s of xenon light exposure [17,19]. Although the current study did not find an improvement in visual acuity or contrast sensitivity within the non-activated-photochromic-CL group, the subjective evaluation might have been enhanced, as per prior reports. These findings, along with the results of this study, suggest that a photochromic CL does not impair visual function within indoor environments, but can enhance visual function in harsh outdoor environments exposed to sunlight and ultraviolet light. This implies that the use of photochromic CLs can provide a comfortable visual experience for individuals whose primary activities take place outdoors and those who primarily reside indoors.

The four limitations of this study are as follows: First, the actual wearing of the photochromic CL has yet to be performed, so the possible changes in tear fluid, corneal shape, and other aspects of CL wearing have not been reproduced. Second, although the CL adheres to the cornea and thus completely covers the cornea from light and ultraviolet light because the simulated lens is worn in the optometric frame, it is exposed to light from outside the frame, and thus does not fully reproduce the transmittance of the photochromic CL. Third, the examinees were relatively young, and the range of age was narrow, since the study participants were recruited from the university students. However, Hammond et al. [20] demonstrated that the subjects wearing photochromic CLs had reduced halos and starbursts, and that these effects became more pronounced with a higher age. Therefore, the improvement of contrast sensitivity might be more prominent when we included not only young but also older subjects in the current study. Fourth, the different usage of the optometers under indoor and outdoor conditions and the differences of the real-world and experimental glare conditions might be biased in the current study, since it was actually difficult to transport the optometers to outdoor conditions, and actual glare conditions were difficult to reproduce. Further studies are required to confirm our current findings.

**Table 3:** Summary of previously reported evaluations for photochromic CL.

Author	Age	Eyes	Refractive Power (D)	Evaluation Item	Significant Differences
Buch, et al. (2022) [14]	32.4 ± 7.9	458	−3.26 ± 1.24	subjective satisfaction (Low Lighting, Bright Outdoor, General Performance)	Yes
Hammond, et al. (2020) [17]	34.9 ± 11.2	34	—	photostress recovery glare disability glare discomfort chromatic contrast	Yes Yes Yes Yes
Kamiya, et al. (2022) [18]	21.7 ± 0.7	82	−2.13 ± 0.92	indoor visual function (visual acuity, kinetic visual acuity, functional visual acuity, ocular higher-order aberrations, contrast sensitivity, satisfaction score)	No Yes Yes No No Yes
Hammond, et al. (2020) [20]	27.1 ± 6.4 51.9 ± 6.9	58	—	photostress recovery, glare disability, glare discomfort, chromatic contrast, vernier acuity	Yes Yes Yes Yes No
Current study	21.3 ± 1.2	33	−1.80 ± 2.82	visual acuity (indoor, outdoor) contrast sensitivity	No No No

## 5. Conclusions

In our simulation study of the photochromic CL using lenses on a spectacle frame indicate that utilization of photochromic CLs may augment visual capacity in adverse conditions, specifically in outdoor settings under bright sunlight when they are in an activated state. Our results also demonstrate that photochromic CLs do not impair visual performance in indoor environments or under intense illumination, even when activated, thereby revealing the widespread suitability of photochromic CLs for individuals who primarily spend time both outdoors and indoors.

## Figures and Tables

**Table 1:** Demographics of the study population.

	Average ± SD
Eyes	33
Age	21.3 ± 1.2
Gender (Male:Female)	13:20
Manifest spherical power (D)	−1.80 ± 2.82
Manifest cylindrical power (D)	0.64 ± 0.59

**Table 2:** Comparison of visual function of non-photochromic-lens group, non-activated-photochromic-CL group, and activated-photochromic-CL group.

	No-Lens	Non-Activated	Activated	*p* Value	No-Lens- Non-Activated	No-Lens- Activated	Non-Activated- Activated
Indoor visual acuity (logMAR)	−0.27 ± 0.05	−0.27 ± 0.06	−0.27 ± 0.06	0.949	—	—	—
Outdoor visual acuity (logMAR)	−0.25 ± 0.07	−0.26 ± 0.06	−0.25 ± 0.07	0.540	—	—	—
Indoor AULCSF	1.48 ± 0.10	1.51 ± 0.08	1.51 ± 0.08	0.355	—	—	—
Outdoor AULCSF	1.53 ± 0.04	1.54 ± 0.05	1.58 ± 0.04	<0.001	0.233	<0.001	0.014
glare AULCSF	1.39 ± 0.08	1.40 ± 0.09	1.42 ± 0.09	0.281	—	—	—

LogMAR = logarithm of the minimal angle of resolution.

## Data Availability

The data that support the findings of this study are available from the corresponding authors, S.S., upon reasonable request.

## Abbreviations

UVR; ultraviolet radiations CL; contact lenses, QOV; quality of vision; AULCSF; area under the log contrast-sensitivity function.
